# Identification of Candidate Circular RNAs Underlying Intramuscular Fat Content in the Donkey

**DOI:** 10.3389/fgene.2020.587559

**Published:** 2020-12-09

**Authors:** Bojiang Li, Chunyu Feng, Shiyu Zhu, Junpeng Zhang, David M. Irwin, Xiaoying Zhang, Zhe Wang, Shuyi Zhang

**Affiliations:** ^1^College of Animal Science and Veterinary Medicine, Shenyang Agricultural University, Shenyang, China; ^2^Department of Laboratory Medicine and Pathobiology, University of Toronto, Toronto, ON, Canada; ^3^Liaoning Province Engineering Center of Modern Agricultural Production Base, Shenyang, China

**Keywords:** circular RNA, expression profile, IMF, regulatory network, donkey

## Abstract

Intramuscular fat (IMF) content is a crucial indicator of meat quality. Circular RNAs (circRNAs) are a large class of endogenous RNAs that are involved in many physiological processes. However, the expression and function of circRNA in IMF in the donkey remains unresolved. Here we performed an expression profiling of circRNAs in the donkey longissimus dorsi muscle and identified 12,727 candidate circRNAs. Among these, 70% were derived from the exons of protein genes. Furthermore, a total of 127 differentially expressed (DE) circRNAs were identified in high (H) and low (L) IMF content groups, including 63 upregulated and 64 downregulated circRNAs. Gene Ontology (GO) and Kyoto Encyclopedia of Genes and Genomes (KEGG) pathway enrichment analysis of the host genes of the DE circRNAs showed that the host genes were enriched in lipid metabolism related GO terms (e.g., fatty acid beta-oxidation using acyl-CoA dehydrogenase and MLL3/4 complex), and signaling pathways (e.g., TGF-beta and lysine degradation signaling pathway). Further analyses indicated that 127 DE circRNAs were predicted to potentially interact with miRNAs, leading to the construction of circRNA-miRNA regulatory network. Multiple circRNAs can potentially function as sponges of miRNAs that regulate the differentiation of adipocytes. Our results provide valuable expression profile information for circRNA in the donkey and new insight into the regulatory mechanisms of circRNAs in the regulation of IMF content.

## Introduction

The donkey (*Equus asinus*) is an important livestock animal in many countries including China and Italy (Polidori et al., 2015; Zhang X. et al., 2019), and plays a crucial role in human agricultural society by providing various products (meat, milk, and leather), acting as a draft force and transportation (Xia et al., 2019). Donkey meat was previously obtained from animals that were slaughtered at the end of their working lives, leading to poor quality meat with bad sensorial and nutritional characteristics (Lorenzo et al., 2014). In recent years, with the increasing mechanization of the world, donkey meat production from young males has improved the quality of the meat and attracted more customers for its consumption (Lorenzo et al., 2014). Therefore, donkey has increasingly become an important meat producing livestock. Donkey meat has recently been recognized as a nutritious food for human consumption, as it contains high-quality protein, vitamins, and minerals (Polidori et al., 2015). For example, the protein content of donkey meat is 22.8%, and the potassium and phosphorus content are 343 and 212 mg per100 g, respectively (Polidori et al., 2008).

IMF, corresponds to the fat within muscles, and its amount is controlled by the number and size of intramuscular adipocytes (Li B. et al., 2018). A previous study indicated that the IMF content plays a key role in various quality traits of meat in many species (Hocquette et al., 2010). Increased levels of IMF content can positively influence sensory quality traits such as tenderness, juiciness, taste, and flavor (Bahelka et al., 2009). IMF content has a relatively high heritability in cattle (*h*^2^ = 0.51) (Nogi et al., 2011) and pig (*h*^2^ = 0.4–0.7) (Pena et al., 2016), which indicates that these animals can be selected for higher IMF content and bred to improve this trait in the next generation. However, there are currently no reports on the heritability of IMF in donkey. IMF content is complex quantitative trait, which is affected by multiple genetic components, environmental conditions, cellular signals and hormones (Cho et al., 2019). Therefore, identifying candidate genes and molecular markers could be useful in selection programs to improve IMF content in the donkey.

To date, groups that have studied donkey meat and carcass performance have focused on these traits (Polidori et al., 2008; De Palo et al., 2016), rather than the mechanisms that control them. Recently, many previous studies have shown that coding and non-coding RNAs (e.g., miRNA and lncRNA) regulate IMF formation in the pig, cattle and chicken (Li B. et al., 2018; Zhang et al., 2018, 2020; Zou et al., 2018). However, these mechanisms of coding or non-coding RNAs in the regulation of IMF deposition in the donkey is unknown. Circular RNAs (circRNAs) are a class of endogenous non-coding RNA that are processed from precursor mRNA (pre-mRNA) by back-splicing with a covalent linkage between the 3′ and 5′ ends (Dong et al., 2017). They are extensively distributed in mammalian cells and lack the typical 5′ caps and 3′ poly(A) tails of coding mRNA (Devaux et al., 2017). Recent studies have provided evidence that the expression levels of circRNAs are highly conserved among species (Legnini et al., 2017). Due to their non-linear structure, circRNAs have higher stability than linear RNAs and are involved in many different physiological processes (Rybak-Wolf et al., 2015; Sun et al., 2019). It has been shown that circRNAs play important roles in biological and physiological processes in livestock (Bahelka et al., 2009; Li H. et al., 2018; Cao et al., 2019). For example, 828 circRNAs were found to be significantly differently expressed between embryonic and adult bovine muscle tissues and that circLMO7 regulates myoblasts differentiation and survival through the sponging of miR-378a-3p (Wei et al., 2017). A recent study by Liu et al. (2018) demonstrated that a large number of circRNAs are significantly differently expressed during adipogenesis in subcutaneous fat in pigs (Liu et al., 2018). However, it is unknown whether circRNAs have any biological role during IMF formation in donkeys. Therefore, here we systematically investigated the expression profile of circRNAs in donkey longissimus dorsi muscles.

First, we performed expression profiling of circRNAs in the donkey longissimus dorsi muscles of donkey using RNA-seq. We then identified differentially expressed circRNAs between high (H) and low (L) IMF content. GO and KEGG analyses were conducted of the host genes of the differentially expressed circRNAs between H and L IMF content. Finally, we predicted that specific miRNAs were adsorbed by the differentially expressed circRNAs, allowing us to construct the circRNA-miRNA regulatory network in IMF.

## Materials and Methods

### Ethics Statement

All animal procedures described in this study were conducted according to the animal husbandry guidelines of Shenyang Agricultural University. The studies in these animals were reviewed and approved by the Ethics Committee and Experimental Animal Committee of Shenyang Agriculture University.

### Animals, Samples Collection, and Phenotypes Measurement

The animals used in this study were derived from 30 Liaoxi donkeys, and provided by Fuxin City Lv Xian Yuan Meat Food Co., Ltd. (Fuxin, China), which had been raised under standard conditions with *ad libitum* access to a mixture of cereal straw and grass hay, maize, bran, peas, minerals, vitamins, and water. At an age of about 15 months, all of the donkeys were slaughtered in the same abattoir (Fuxin, China). Longissimus dorsi muscle was collected and used for the measurement of IMF content and RNA extraction. Tissue samples for RNA extraction were immediately snap-frozen in liquid nitrogen, and then stored at −80 °C until use. IMF content of the longissimus dorsi muscle was measured using the Soxhlet extraction method as described previously (Li B. et al., 2018). Of the 30 samples tested, the three with the H IMF content and the three with the L IMF content were chosen for RNA extraction. The IMF content of the H and L groups were 6.34% (SEM = 0.47%) and 3.04% (SEM = 0.12%), respectively, which was significantly different (*P* ≤ 0.05), however, the body weights of the animals in these two groups were not different.

### RNA Isolation, Library Construction, and RNA Sequencing (RNA-Seq)

Total RNA was isolated from each sample using TRIzol (Invitrogen, Carlsbad, CA, United States) according to manufacturer’s protocol. RNA concentration and purity were assessed using a NanoDrop 2000 spectrophotometer (Thermo Fisher Scientific, Waltham, MA, United States). The integrity of the RNA was assessed using an Agilent 2,100 Bioanalyzer (Agilent Technologies, Santa Clara, CA, United States). Ribosomal RNA (rRNA) was removed using a Ribo-Zero Magnetic Gold Kit (Epicenter, Madison, WI, United States). The linear-stranded RNA was removed by RNase R (Epicenter, Madison, WI, United States). Sequencing libraries were generated using NEBNext^®^ Ultra^TM^ Directional RNA Library Prep Kit for Illumina (NEB, Ipswich, United States) following manufacturer’s recommendations. Sequencing of the libraries was performed on an Illumina Novaseq 6,000 system with PE150 sequencing mode by Novogene Co., Ltd. (Beijing, China).

### CircRNA Sequencing Analysis

Clean reads were obtained by removing those composed of adapters, contain ploy-N, and low-quality sequences (containing more than 50% low-quality bases) from the raw data. All of the downstream analyses were based on the clean high-quality data. Clean reads were then aligned with the donkey reference genome (ASM303372v1) using the Burrows-Wheeler Aligner (BWA)-MEM (Li and Durbin, 2009). CIRI2 (Gao et al., 2018) software was applied to obtain back-spliced junction reads for circRNA prediction. The expression levels of individual circRNA were calculated as RPM (reads per million mapped reads). DESeq2 (Love et al., 2014) was used to identify differentially expressed (DE) circRNAs between the H and L groups. We defined circRNAs that had a fold change ≥ 2 or < 0.5 with a Benjamini-Hochberg method corrected *p* ≤ 0.05 between the two groups as significant differentially expressed circRNA.

### GO and KEGG Pathway Enrichment Analysis

Gene Ontology (GO) enrichment analysis of the host genes of all differentially expressed circRNAs was performed by the clusterProfiler R package using default parameters (Yu et al., 2012). GO terms with a Benjamini-Hochberg method corrected *p* < 0.05 were considered significantly enriched. We used KOBAS software (Wu et al., 2006) with default parameters to test the statistical enrichment of all host genes of the differentially expressed circRNAs in KEGG pathways. Pathways with a Benjamini-Hochberg method corrected *p <* 0.05 were considered significantly enriched.

### Prediction of CircRNA-miRNA Interactions

We used miRanda software (John et al., 2004) with “-sc 140 -en -10 -scale 4 -strict” to identify putative circRNA-miRNA interactions and Cytoscape software (Shannon et al., 2003) was used to construct the circRNA-miRNA networks.

### Quantitative Reverse-Transcription PCR Analysis

Total RNA was extracted from longissimus dorsi muscle used in the RNA-seq using TRIzol (Invitrogen, Carlsbad, CA, United States), and then reverse-transcribed into complementary DNA (cDNA) using Primescript RT Master Kit (Takara, Dalian, China) according to the manufacturer’s instructions. According to the method of selecting DE circRNA in other studies (Zhang G. et al., 2019; Tian et al., 2020), we randomly select 5 DE circRNAs from the up-regulated and down-regulated DE circRNAs using sample function in R software, respectively. Primers for the differentially expressed circRNAs were designed using primer5 (Singh et al., 1998). All the primers used are listed in Supplementary Table S1. Quantitative Reverse-Transcription PCR (qRT-PCR) was performed with AceQ qPCR SYBR Green Master Mix (Vazyme, Nanjing, China) in a reaction volume of 20 μL. The cycling parameters were as follows: 95°C for 5 min, followed by 40 amplification cycles, each at 95°C for 10 s, then 60°C for 30 s. All reactions were performed in triplicate for each sample. Relative expression levels of the differentially expressed circRNAs were calculated by the 2^–ΔΔ*Ct*^ method as reported previously (Wei et al., 2017; Cheng et al., 2019). The glyceraldehyde-3-phosphate dehydrogenase (GAPDH) gene was used as a reference to normalize the relative expression of the circRNAs.

### Statistical Analyses

Statistical analyses were conducted using SPSS 20.0 (SPSS Inc., Chicago, IL, United States). Statistical significance of the difference between the two groups was calculated using a Student’s *t*-test. A value of *p* < 0.05 was considered to represent a statistically significant difference.

## Results

### Characterization of CircRNAs in Longissimus Dorsi Muscles

**FIGURE 1:**
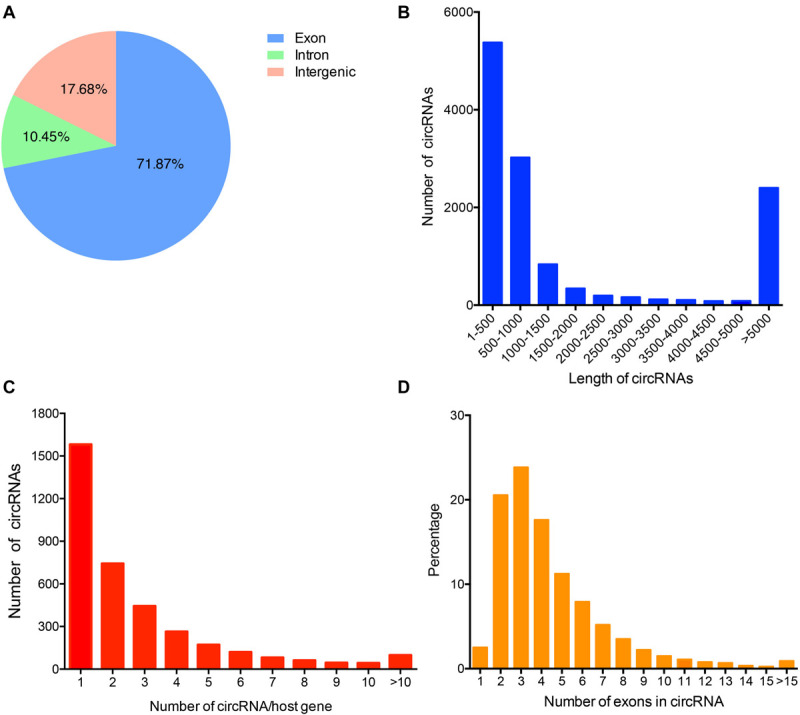
Characteristics of the circRNAs identified in donkey muscle tissue. **(A)** Genomic origin of the identified circRNAs. **(B)** Length distribution of the identified circRNAs. **(C)** Distribution of the number of circRNA per gene. **(D)** The percentage of circRNAs was calculated based on the number of exons each circRNA in the exon-derived circRNAs.

To identify the circRNAs expression profile in donkey longissimus dorsi muscles, we performed RNA-seq and mined the data for circRNAs. In total, 663,324,766 raw reads (327,437,586 for H and 335,887,180 for L group) were generated from the six sequencing libraries (Supplementary Table S2). After removing low-quality, poly-N containing, and adapter-containing reads from the raw reads, 318,388,574 and 326,916,290 clean reads were obtained, for the H and L groups, respectively (Supplementary Table S2). An average of 82.49% (range: 78.58–85.87%) of the reads were mapped to the donkey genome (ASM303372v1) (Supplementary Table S3). A total of 12,727 circRNAs that were supported by at least two junction reads were identified from longissimus dorsi muscle and spliced from 3,251 genes (Supplementary Table S4). Approximately 70% of the circRNAs from both groups were derived from the exons of protein genes, while some of them were from intronic or intergenic regions (Figure 1A). The lengths of most of the identified circRNAs were less than 1,000 nucleotides (nt) (Figure 1B). Among the circRNA-producing genes, most host genes preferred to generate a single circRNA instead of multiple circRNA (Figure 1C). The majority of the identified circRNA species that originate from protein-coding genes contain two or three exons (Figure 1D).

### Identification of DE CircRNAs Between H and L IMF Muscles

**FIGURE 2:**
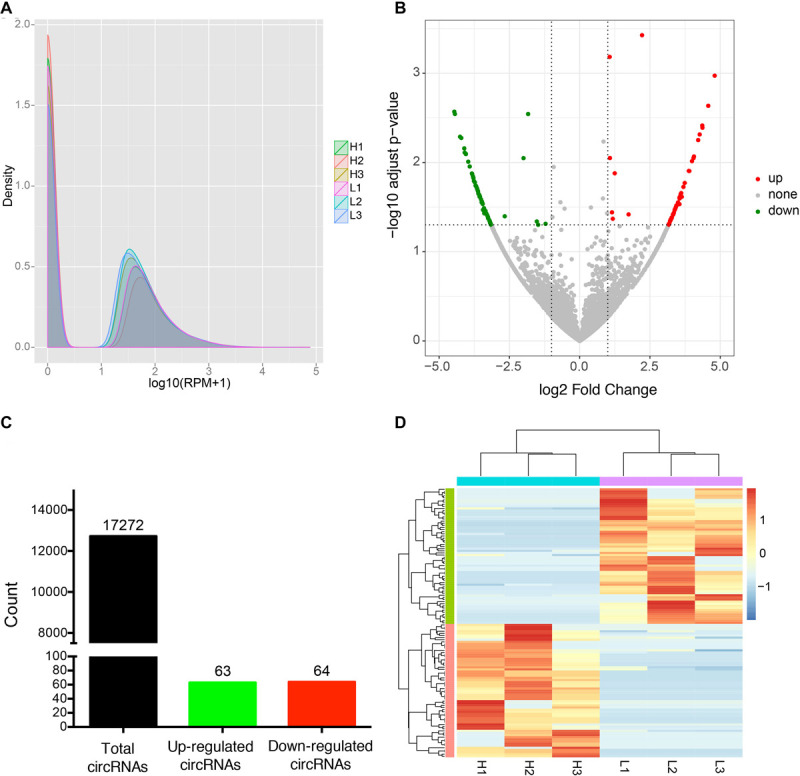
Differential expression analysis of circRNAs between H and L IMF groups. **(A)** Density plot of the expression density distribution of circRNAs in each sample. **(B)** Volcano plot of all DE circRNAs between the H and L IMF groups. *X*-axis represents the value of log2 (H/L) and the *Y*-axis represents the value of –log10 (*p*-value). **(C)** Number of total, up-regulated and down-regulated circRNAs between the H and L IMF groups. **(D)** Clustered heatmap of the expression patterns of the DE circRNAs from three H and three L IMF samples.

To identify candidate circRNAs affecting IMF content in donkey longissimus dorsi muscle, we calculated the expression level of each identified circRNAs and tested for differential expression of circRNAs between the H and L IMF content groups. Our results indicate that the density distribution of circRNA expression was not significantly different between the H and L IMF content groups (Figure 2A), however, 127 DE circRNAs were identified, including 63 upregulated and 64 downregulated circRNAs in the H IMF content group (Figures 2B,C). Detailed information on each DE circRNAs is provided in Supplementary Table S5. Among the candidates, novel_circ_0000323 was the most upregulated circRNA and novel_circ_0000319 was the most downregulated circRNA in the H IMF content group (Supplementary Table S5). Figure 2D shows a heatmap of the expression pattern of the DE circRNAs from the six samples, which indicates that the samples from the H IMF content group could be clearly distinguished from those from the L IMF content group.

### Experimental Validation of the DE CircRNAs

**FIGURE 3:**
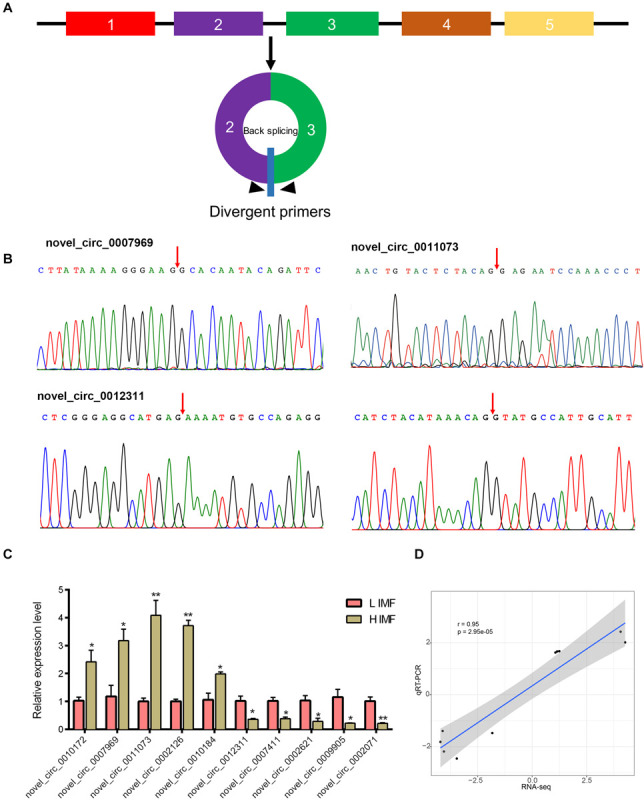
Validation of DE circRNAs by qRT-PCR. **(A)** Schematic of the divergent primer design for circRNAs. **(B)** Representative PCR products sequenced to validate the backsplice sequence of the circRNAs. Arrow represents the backsplice junction. **(C)** Expression level of DE circRNAs in H and L IMF samples were determined by qRT-PCR. *p < 0.05; **p < 0.01, two-tailed *t*-test. **(D)** Pearson correlation coefficient of the log2(fold change) data between qRT-PCR and RNA-Seq.

To verify the reliability of the RNA-seq data, we use qRT-PCR to detect the expression levels of DE circRNAs. We tested the expression of randomly selected five upregulated circRNAs (novel_circ_0010172, novel_circ_0007969, novel_circ_0011073, novel_circ_0002126, and novel_circ_0010184) and downregulated circRNAs (novel_circ_0012311, novel_circ_0007411, novel_circ_0002621, novel_circ_0009905, and novel_circ_0002071). Divergent primers for each circRNA were designed to amplify the back-splice sequences (Figure 3A). PCR products of the divergent primers for each circRNA were confirmed by Sanger sequencing (Figure 3B and Supplementary Figure S1). Moreover, qRT-PCR results indicate that these circRNAs were significantly differentially expressed between H and L IMF groups (*p* < 0.05; Figure 3C), and the Pearson correlation coefficient of the log2(fold change) data between the qRT-PCR and RNA-Seq was 0.95 (*p* < 0.05; Figure 3D), suggesting that the DE circRNAs identified by RNA-Seq were reliable.

### Enrichment Analysis of the Host Genes of DE CircRNAs

**FIGURE 4:**
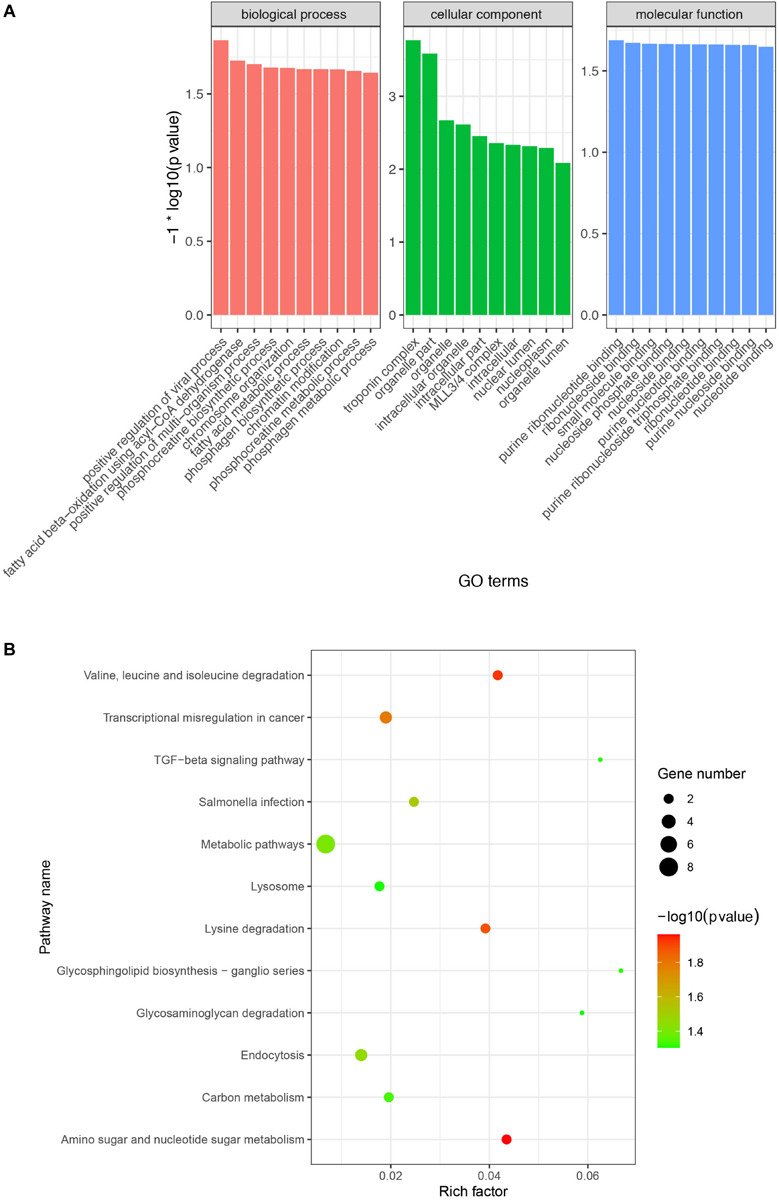
Gene Ontology (GO) and KEGG enrichment analysis of host genes of the DE circRNAs. **(A)** Most enriched GO terms in biological process, cellular component, and molecular function. *X*-axis represents GO terms and the *Y*-axis represents the value of -log10 (*p*-value). **(B)** Significantly enriched signaling pathways of the host genes of the DE circRNAs. The *X*-axis represents rich factor and the *Y*-axis represents pathway. Size and color of the bubble represent the number of host genes enriched in the pathway and enrichment significance, respectively.

A previous study demonstrated that circRNA abundance is negatively correlated with their linear host gene mRNA and that there is a competition between pre-mRNA splicing and circRNA production (Ashwal-Fluss et al., 2014). To explore and analyze the potential biological function of these DE circRNAs, we performed GO and KEGG enrichment analysis of the host genes of the DE circRNAs. GO enrichment analysis indicated that 48, 37, and 36 GO terms were significantly enriched in biological process, cellular component, and molecular function, respectively (Supplementary Table S6). Among the most enriched GO terms, some of them were associated with lipid metabolism (e.g., fatty acid beta-oxidation using acyl-CoA dehydrogenase and MLL3/4 complex) (Figure 4A). Moreover, the KEGG enrichment analysis indicated that the host genes of the DE circRNAs were significantly enriched in 12 pathways, including related to lipid metabolism (e.g., TGF-beta and lysine degradation signaling pathway) (Figure 4B).

### Putative Functions of the DE CircRNAs as MiRNA Sponges

**FIGURE 5:**
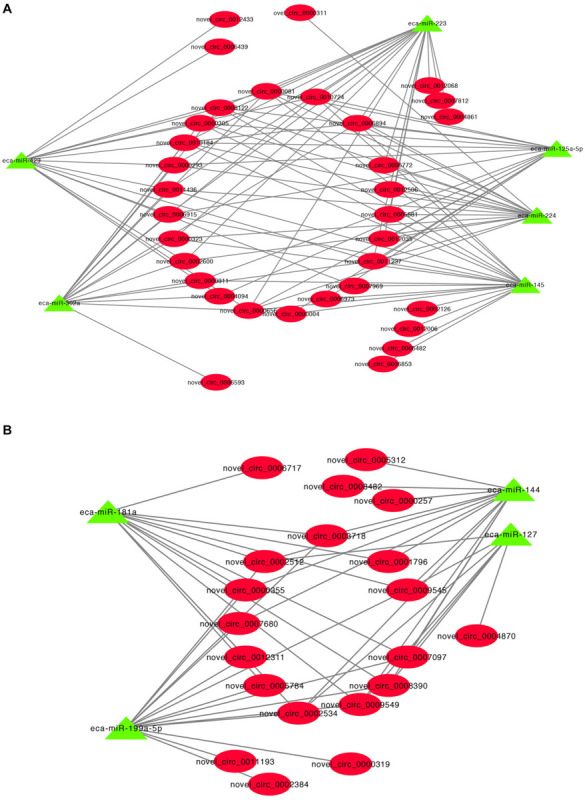
CircRNA-miRNA regulatory network analysis. **(A)** Upregulated circRNA regulatory networks and **(B)** downregulated circRNAs regulatory networks.

A previous study has shown that circRNA can act as miRNA sponges by acting as binding sites (Li X. et al., 2018). Some microRNAs (miRNAs) function as stimulators or inhibitors in adipocyte differentiation (Arner and Kulyte, 2015). To determine whether the DE cricRNA identified in this study can potentially function as miRNA sponges to regulate adipogenesis, we tested the ability of the DE circRNAs to bind to miRNAs. The circRNA-miRNA regulatory networks showed that 17,088 circRNA-miRNA interactions could be predicted based on 127 DE circRNAs and 690 miRNAs (Supplementary Table S7). Interestingly, we found that many of the upregulated circRNAs potentially function as sponges for many miRNA genes associated with adipogenesis such as miR-429, miR-224, miR-125a-5p, miR-223, miR-145, and miR-302a (Figure 5A). Additionally, many of the downregulated circRNAs also potentially bind miRNAs such as to miR-181a, miR-144, miR-199a-5p, and miR-127 (Figure 5B).

## Discussion

With the advancement of high-throughput sequencing technologies and bioinformatics, a large number of circRNAs have been identified in the human (Li S. et al., 2018), mouse (Legnini et al., 2017), chicken (Shen et al., 2019), pig (Hong et al., 2019; Li et al., 2020), cattle (Li H. et al., 2018), and sheep (Li X.Y. et al., 2019) genomes. Recently, the circRNA database CircAtlas^1^ has integrated over one million circRNAs from 6 species (human, macaca, mouse, rat, pig, and chicken) (Wu et al., 2020). However, the expression profiles and biological characteristics of circRNAs in the donkey has not been examined. Here we performed a comprehensive profile of circRNAs in the donkey using RNA-seq and identified 12,727 unique circRNAs in longissimus dorsi muscle samples. These results provide the foundation for establishing a donkey circRNA database and allowing future research on the potential role of circRNA in muscle development in the donkey. In this study, the major circRNAs were found to be derived from back-splicing of exons, which is consistent with previous studies in pig (Liang et al., 2017), chicken (Ouyang et al., 2017), and cattle (Wei et al., 2017) muscle. This result suggests that the circRNAs are highly similar in distribution pattern and evolutionarily conserved among different species. Moreover, our results show that the identified circRNAs contain multiple exons, with most containing two or three exons, which is similar to the circRNAs in many other species (Zhang et al., 2014; Wei et al., 2017), suggesting many host preferentially produce two or three circRNAs in donkey.

Previous studies have demonstrated that circRNAs are a large class of widespread and diverse endogenous RNAs in animals that play critical roles in many physiological and pathological processes, including fat (Jiang et al., 2020), muscle (Zhang P. et al., 2019), and cancer (Xie et al., 2020) development. For example, Jiang et al. (2020) characterized the expression profile of circRNA in fat tissue during developmental stages of the calf and adult cow and demonstrated that the circFUT10 regulates adipocyte cell proliferation and differentiation by sponging the miRNA let-7c (Jiang et al., 2020). Moreover, several studies have found that circRNAs are involved in muscle traits in the pig (Liang et al., 2017; Sun et al., 2017) and chicken (Chen et al., 2019). Recent studies have shown a potential role for circRNAs in IMF deposition in the yak based on the differentially expression of circRNAs between muscle and adipose tissue (Wang et al., 2020). In this study, we obtained the expression profiles of circRNA from muscle with differing IMF content, and identified 127 differentially expressed circRNAs between three H and L IMF samples. However, the expression level of these DE circRNAs in a large number of individuals are still needed to be confirmed. Furthermore, 10 DE circRNAs detected by qRT-PCR are significantly differentially expressed between H and L groups, indicating the DE circRNA results are reliable. These results suggest that these DE circRNAs play an important role in IMF deposition, and that circRNAs may affect livestock meat quality through the formation of IMF.

Since circRNAs are produced from precursor mRNA (pre-mRNA) by back-splicing, the production of circRNA can reduce the amount of linear transcripts by competing with linear splicing (Ashwal-Fluss et al., 2014; Liu et al., 2018). This suggests that circRNAs can act in an important role under physiological conditions to regulate the expression of their host genes. In this study, GO enrichment of host genes of DE circRNAs yielded some GO terms that are involved in lipid metabolism including MLL3/4 complex, fatty acid beta-oxidation using acyl-CoA dehydrogenase, fatty acid metabolic process, methyltransferase complex and histone deacetylase binding. Histone methyltransferases MLL3/4 play a crucial role in adipogenesis (Lee et al., 2020) and the deletion of MLL3 in mice results in significantly decreased amounts of white fat (Lee et al., 2008). For example, novel_circ_0009716, a down-regulated circRNA from the H IMF group, is derived from the histone mono-methyltransferase MLL4 (KMT2D), suggesting that it can regulate IMF formation through the MLL3/4 complex. Moreover, novel_circ_0007020 is derived from the MIER1 gene, which is enriched in the histone deacetylase binding GO term. A previous study indicated that MIER1 can bind to BAHD1 to form a hub for histone deacetylases and methyltransferases that are involved in energy metabolism (Lakisic et al., 2016). Therefore, novel_circ_0007020 might be involved in IMF formation by repressing the levels of its linear mRNA for MIER1. In addition, we found that host genes of the differently expressed circRNAs were significantly enriched in 12 pathways, including related to lipid metabolism (e.g., TGF-beta and lysine degradation signaling pathway). TGFβ and its downstream effector have been reported to play important roles in regulating glucose and energy homeostasis (Yadav et al., 2011). Previous study has shown that increased IMF deposition is due to lysine restriction in pig diets (Madeira et al., 2013). These evidences indicate that DE cricRNAs can regulate TGF-beta and lysine degradation signaling pathway by competing with host mRNA splicing, which ultimately affect IMF deposition. Together, all these results suggest that these differentially expressed circRNAs affect the physiological functions of their host genes leading to the regulation of IMF deposition.

It has been shown that circRNA can act as competing endogenous RNAs (ceRNAs) to sponge miRNAs and prevent them from binding and suppressing their target mRNAs (Chen, 2020). For example, circHIPK2 regulates astrocyte activation, via regulation of autophagy and endoplasmic reticulum (ER) stress, through targeting of MIR124-2HG and SIGMAR1 (Huang et al., 2017). We identified 17,088 circRNA-miRNA interactions in this study and these results suggest that DE circRNAs have a regulatory role in IMF deposition via the sequestration of miRNAs. MicroRNAs (miRNAs) are a class of small non-coding RNA that regulate target gene expression, and can stimulate or inhibit the differentiation of adipocytes in adipose tissue (Arner and Kulyte, 2015). Our current study revealed that many DE circRNAs may act as endogenous sponges of miRNAs including miR-429 (Peng et al., 2016), miR-224 (Zhang Y. et al., 2019), miR-125a-5p (Du et al., 2018), miR-223 (Li F. et al., 2019), miR-145 (Guo et al., 2012), miR-302a (Jeong et al., 2014), miR-181a (Zhang Z. et al., 2019), miR-144 (Shen et al., 2018), miR-199a-5p (Shi et al., 2014), and miR-127 (Gao et al., 2019) that are involved in the regulation of adipogenesis. miR-125a-5p is negatively involved with IMF content by targeting *KLF13* and *ELOVL6* (Du et al., 2018). We predicted that fifteen upregulated DE circRNA, such as novel_circ_0010184, can able to bind miR-125a-5p. Moreover, novel_circ_0010184 was upregulated in H IMF content compared to L IMF content using qRT-PCR in this study. Therefore, these results suggest that these circRNAs might be a sponge of miR-125a-5p and promote increases in IMF content. A downregulated DE circRNA, novel_circ_0012311, might function as sponge for miR-181a that facilitates porcine preadipocyte differentiation by targeting TGFBR1, indicating that novel_circ_0012311 might inhibit IMF preadipocyte differentiation through the miR-181a/TGFBR1 axis in donkey. Consequently, our results suggest that DE circRNAs have important roles in the regulation of IMF adipogenesis by acting as miRNA sponges to inhibit the miRNA targeting of genes.

## Conclusion

We identified 12,727 circRNAs in the donkey longissimus dorsi muscle, thus, expanding our understanding of the complexity of the donkey transcriptome. The host genes were enriched in lipid metabolism related GO terms and signaling pathways, and DE circRNAs act as adipogenesis related miRNAs sponge, which provide insight into our understanding of the functions of circRNA in IMF content. Our results provide evidence that circRNAs have an important regulatory role in IMF content and this information might be useful for future research into circRNA and the regulation of IMF deposition.

## Data Availability Statement

The data used in our study have been deposited in NCBI SRA (accession codes PRJNA647167).

## Ethics Statement

The animal study was reviewed and approved by the Ethics Committee and Experimental Animal Committee of Shenyang Agriculture University. Written informed consent was obtained from the owners for the participation of their animals in this study.

## Author Contributions

SZha conceived the study. BL, CF, SZhu, JZ, and XZ conducted the experiments. ZW analyzed the data. BL wrote the manuscript. DI revised the manuscript. All authors contributed to the article and approved the submitted version.

## Conflict of Interest

The authors declare that the research was conducted in the absence of any commercial or financial relationships that could be construed as a potential conflict of interest.

## Footnotes

1 http://circatlas.biols.ac.cn/
