# Supplementary material for: Identification of Candidate Circular RNAs Underlying Intramuscular Fat Content in the Donkey
Source: Front Genet. 2020 Dec 9;11:587559. doi: 10.3389/fgene.2020.587559 (PMC7793956; doi:10.3389/fgene.2020.587559)
Supplement: Supplementary file 1 [file Data_Sheet_1.ZIP › supplememtary/Supplementary Table S1 Primers used in this study.docx]

**Supplementary Table S1 Primers used in this study**

| Gene name | Primer sequences |
| --- | --- |
| novel_circ_0010172 | F: CAGGGGAGTACAGCTGTGAG  R: TCCTTGAACCACGTCACCTC |
| novel_circ_0007969 | F: ACCCTCAGCCACAACCTTAT  R: CTTGTTCCTGCTGACACTGG |
| novel_circ_0011073 | F: GTCAGTGTGAAGGGGAGGTT  R: CCGACGCAGGACAGATATGA |
| novel_circ_0002126 | F: AGAGCCTGCAATCTGTGGG  R: AGCAATTCTTTTCCCACGTGA |
| novel_circ_0010184 | F: CAGACACTGGGGACTACTCG  R: CCTCTTCCTTCCTCAGACCC |
| novel_circ_0012311 | F: CGAGAGTACAGAGGCACCAA  R: ACAGGCGGTTTGATGTGTTC |
| novel_circ_0007411 | F: ACTGGGTCCTTAGCACTGTT  R: GGGAAAAGTACGAGGTGAGC |
| novel_circ_0002621 | F: GCCATGAATGCCCAACAAGA  R: TGGCTGTCATCCTTCCCAAT |
| novel_circ_0009905 | F: GAGATTCAAGCGTGGTGTGG  R: TGGGCTGGTAAATATTACACGC |
| novel_circ_0002071 | F: AAAACAAACCCCAGTGCCTG  R: CACGGATCTCAGTCACTCCA |
| GAPDH | F: CTTGTCTCCCTCAGATTTGGC  R: AAGGGGTCATTGATGGCGAC |
