# Supplementary material for: Identification of Candidate Circular RNAs Underlying Intramuscular Fat Content in the Donkey
Source: Front Genet. 2020 Dec 9;11:587559. doi: 10.3389/fgene.2020.587559 (PMC7793956; doi:10.3389/fgene.2020.587559)

>novel_circ_0010172

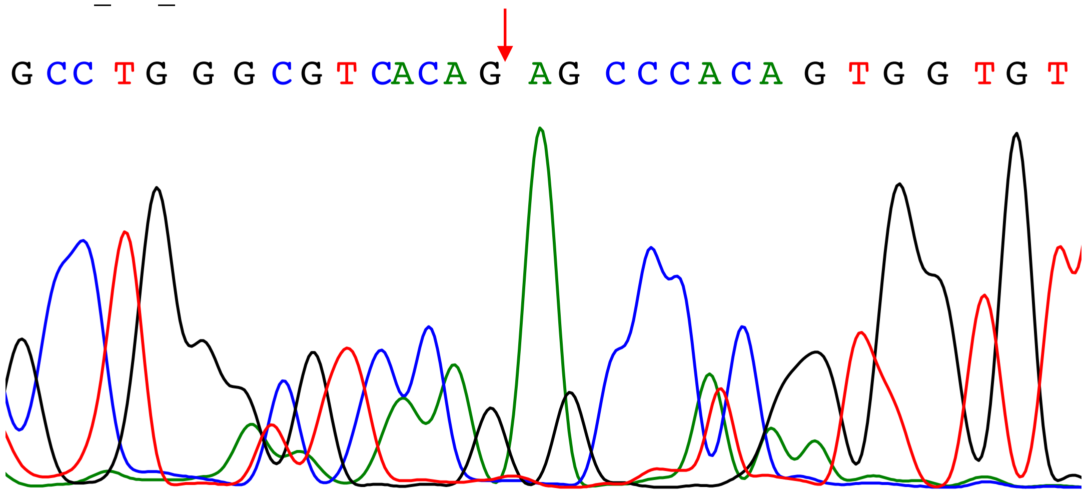

>novel_circ_0002126

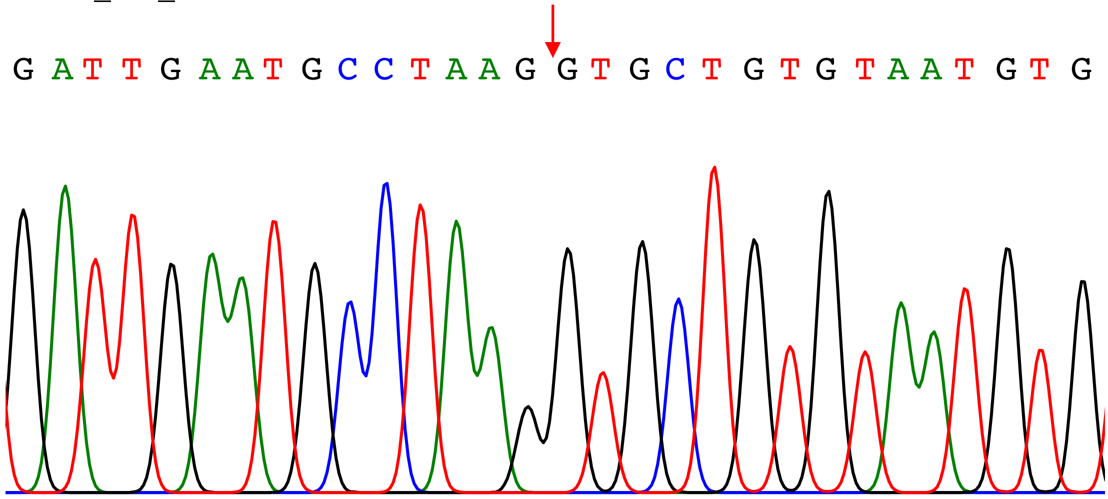

>novel_circ_0010184

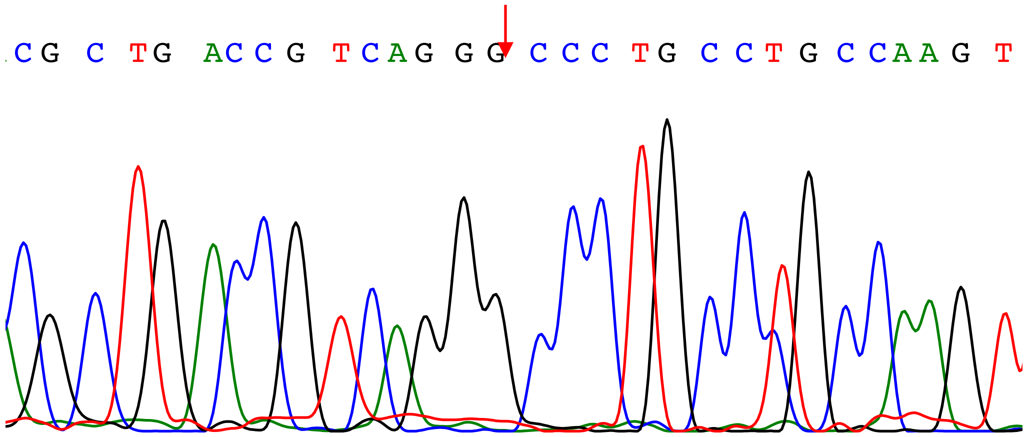

>novel_circ_0007411

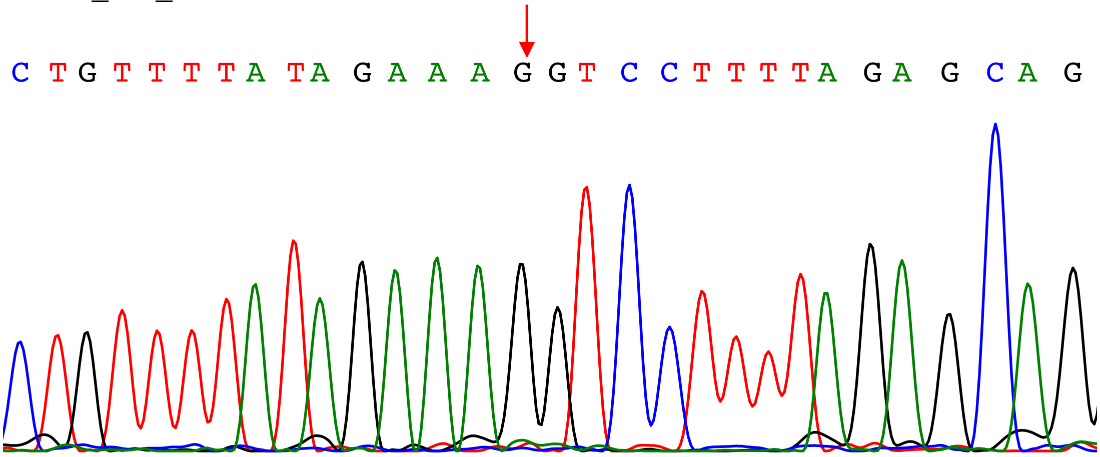

>novel_circ_0002621

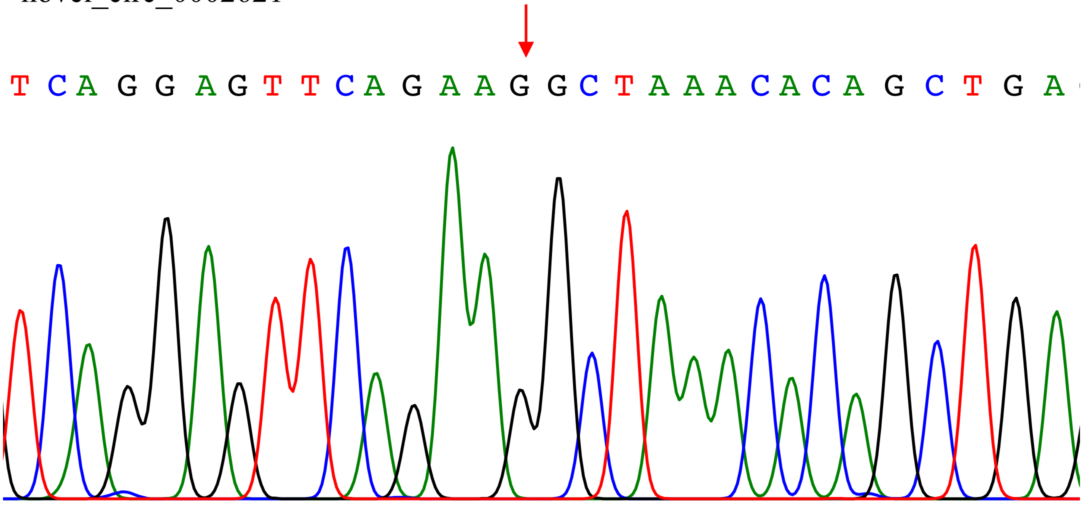

>novel_circ_0002071

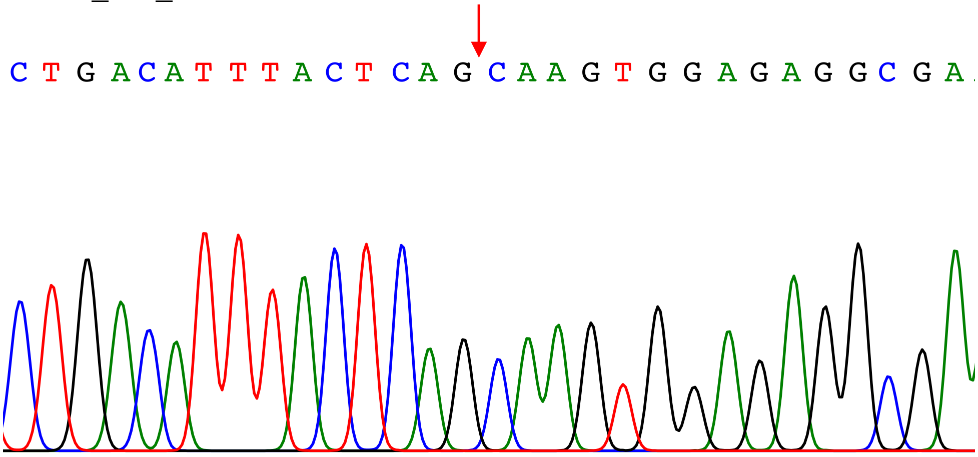

Supplement: Supplementary file 1 [file Data_Sheet_1.ZIP › supplememtary/Supplementary Figure S1 Sanger sequencing of circRNAs.pdf]
